# Neutrophils Which Migrate to Lymph Nodes Modulate CD4^+^ T Cell Response by a PD-L1 Dependent Mechanism

**DOI:** 10.3389/fimmu.2019.00105

**Published:** 2019-01-29

**Authors:** Sofía D. Castell, María F. Harman, Gabriel Morón, Belkys A. Maletto, María C. Pistoresi-Palencia

**Affiliations:** ^1^Departamento de Bioquímica Clínica, Facultad de Ciencias Químicas, Universidad Nacional de Córdoba, Córdoba, Argentina; ^2^Consejo Nacional de Investigaciones Científicas y Técnicas, Centro de Investigaciones en Bioquímica Clínica e Inmunología, Córdoba, Argentina

**Keywords:** immune complex, neutrophils, PD-L1, CD4^+^ T cells response, modulation, lymph nodes

## Abstract

It is well known that neutrophils are rapidly recruited to a site of injury or infection and perform a critical role in pathogen clearance and inflammation. However, they are also able to interact with and regulate innate and adaptive immune cells and some stimuli induce the migration of neutrophils to lymph nodes (LNs). Previously, we demonstrated that the immune complex (IC) generated by injecting OVA into the footpad of OVA/CFA immunized mice induced the migration of OVA^+^ neutrophils to draining LNs (dLNs). Here we investigate the effects of these neutrophils which reach dLNs on CD4^+^ T cell response. Our findings here strongly support a dual role for neutrophils in dLNs regarding CD4^+^ T cell response modulation. On the one hand, the CD4^+^ T cell population expands after the influx of OVA^+^ neutrophils to dLNs. These CD4^+^ T cells enlarge their proliferative response, activation markers and IL-17 and IFN-γ cytokine production. On the other hand, these neutrophils also restrict CD4^+^ T cell expansion. The neutrophils in the dLNs upregulate PD-L1 molecules and are capable of suppressing CD4^+^ T cell proliferation. These results indicate that neutrophils migration to dLNs have an important role in the homeostasis of adaptive immunity. This report describes for the first time that the influx of neutrophils to dLNs dependent on IC presence improves CD4^+^ T cell response, at the same time controlling CD4^+^ T cell proliferation through a PD-L1 dependent mechanism.

## Introduction

Neutrophils are critical effector cells of the innate immune system and rapidly respond to tissue injury and infection. The main role of neutrophils in an inflammatory immune response has been associated with the elimination of pathogens by phagocytosis, the generation of reactive oxygen species, the formation of extracellular traps, and the release of granule enzymes, and inflammatory mediators (1, 2).

However, there is increasing evidence that neutrophils are versatile cells with the ability to interact with different cells, from not only the innate but also the adaptive immune system (3, 4). It has been reported that neutrophils can directly or indirectly modulate lymphocyte functions (5). The regulatory mechanisms of neutrophils induce the functional maturation of B cells (6, 7) and the regulation of T cell activation (8).

Neutrophils can affect CD4^+^ T cell response directly by releasing soluble mediators that regulate their priming (9). They also induce the activation of dendritic cells (DCs) and thus indirectly contribute to the subsequent T cell response (10, 11). Other studies revealed that they are able to act as antigen-presenting cells (APCs) (12, 13), and that neutrophils can suppress CD4^+^ T cell response by secreting IL-10 (14, 15), inhibiting DC functions (16), and through the expression of programmed death ligand 1 (PD-L1) (17–19).

Neutrophils have the capacity to migrate to lymph nodes (LNs) in response to different stimuli (20). Indeed, we previously reported that the injection of ovalbumin (OVA) into the footpad of previously OVA/Complete Freund's adjuvant (CFA)-immunized mice generates the formation of immune complexes (IC), and as a consequence the main OVA^+^ cells in footpads and draining popliteal LNs are neutrophils (21). We also showed that neutrophils can enter LNs of OVA/CFA immunized mice not only via lymphatic vessels but also from blood across high endothelial venules, and we identified the homing molecules required for their trafficking into LNs (22). With all this in mind, we hypothesized that those neutrophils must be involved in the modulation of the adaptive immune response in LNs.

The aim of this work was to evaluate the impact of neutrophils which reach dLNs on CD4^+^ T cell response in immunized mice after OVA footpad challenged. The data presented show that the influx of OVA^+^ neutrophils to LNs induced CD4^+^ T cell activation and proliferation and the production of IL-17 and IFN-γ cytokines. We also found that these neutrophils express PD-L1 and restrict CD4^+^ T cell proliferation. Our results establish that neutrophils have an important role in the homeostasis of adaptive immunity in LNs, promoting the activation, and expansion of CD4^+^ T cells but also controlling the CD4^+^ T cell response.

## Materials and Methods

### Mice and Treatment

Wild-type (WT) C57BL/6 mice were purchased from the Fundación Facultad de Ciencias Veterinarias (Universidad Nacional de La Plata, La Plata, Argentina). PD-L1^−/−^ mice were provided by Dr. Halina Offner (Oregon Health and Science University, Portland, OR, USA). OT-II mice, which express a transgenic TCR designed to recognize OVA residues 323–339 in the context of H-2 I-A^b^ (23), were provided by Dr. F.A. Goldbaum (Fundación Instituto Leloir, Buenos Aires, Argentina). Mice were maintained in our animal facility until use, following the standards of the Guide to the Care and Use of Experimental Animals published by the Canadian Council on Animal Care, with the assurance number A5802–01 assigned by the Office of Laboratory Animal Welfare (NIH). Our Institutional Animal Experimentation Committee approved the animal handling and experimental procedures (Approval Numbers 834/2015 and 746/2018).

Female 8 to 10-week-old mice were subcutaneously immunized with 60 μg OVA (Fraction V; Sigma Aldrich) emulsified in CFA (Sigma Aldrich) and 15 days later with 60 μg OVA emulsified in Incomplete Freund's adjuvant (IFA) (Sigma Aldrich). Each animal was immunized with an entire dose (0.5 ml/animal/dose) distributed at three sites in the back region. Ten days after the second immunization, mice were injected in the hind footpad with 30 μg OVA, or 30 μg OVA conjugated with FITC, following a previously described protocol (24), or 6 × 10^6^ IC-stimulated purified neutrophils or with saline solution (SS) as control.

### Specific Antibody Detection Assay

Specific antibodies against OVA were determined by ELISA following a previously described protocol (25). Briefly, 96-well flat-bottom plates were coated with OVA (1 μg/well) and later were incubated with serial dilutions of mice plasma. HRP-conjugated anti-mouse polyclonal IgG (Sigma Aldrich), IgG1 (X56) and IgG2a/c (R19-15) (both from Becton Dickinson) were used as detection antibodies. Titers were calculated as the reciprocal of the last plasma dilution that yielded an absorbance at 490 nm above that of twice the mean value of blank.

### Cells Preparation and Flow Cytometry

Three to 56 h after footpad injection, mice were sacrificed, and blood, bone marrow (BM), and LNs were removed to obtained single-cell suspensions. Red blood cells were lysed using RBC lysing buffer (Sigma Aldrich). For surface staining, cells were pre-incubated with anti-CD16/36 (2.4G2) for 15 min at 4°C, and then stained for 30 min at 4°C with the following fluorochrome-labeled antibodies: anti-mouse Ly6G (1A8), CD4 (RM4-5), CD62L (MEL14), CD11b (M1/70), PD-L1 (MIH5), and TCR Vβ 5.1/5.2 (MR9-4) from BD Bioscience; anti-mouse CD3 (145-2C11), CD11c (N418), CD44(IM7), and CD69 (HI.2F3) from eBioscience. For intracellular detection of cytokines, LN cells (10^6^ cell/well) were incubated with 0.65 μl/ml StopGolgi Monensin (BD Biosciences) at 37°C for 4 h. Then, cells were stained for surface markers before being fixed and permeabilized using the BD Cytofix/Cytoperm™ Plus Kit (BD Biosciences) following the manufacturer's instructions. Finally, cells were stained with fluorochrome-labeled anti-mouse TNF (MP6-XT22), IL-17 (11B7), IFN-γ (XMG1.2), or isotype-matched control antibody (all from eBioscience) for 30 min at 4°C. For intracellular staining of Ki67, LN cells (10^6^ cell/well) were stained for surface markers, then fixed and permeabilized with the Transcription Factor Staining Buffer Set (eBioscience) in accordance with the manufacturer's instructions. Then cells were stained with fluorochrome-labeled anti-mouse Ki67 (SolA15) from eBioscience for 30 min at 4°C. In all cytometry assays, fixable viability dye (eBioscience) was used to identify live cells. Data acquisition was performed on a FACS II cytometer (BD Bioscience) and analyzed using FlowJo software (Tree Star Inc., Ashland OR).

### Lymph Nodes Cells Culture

Cell suspensions obtained from LNs were cultured in GIBCO® RPMI 1640 medium (Life Technologies) supplemented with 10% heat-inactivated fetal bovine serum (NATOCOR), 2 mm GIBCO® Glutamax, 100 U/ml Penicillin with 100 μg/ml Streptomycin (both from Life Technologies) and 50 μm 2-mercaptoethanol (Sigma Aldrich) at 37°C in a 5% CO_2_ humidified incubator. When indicated, cells were stimulated with 100 μg/ml OVA, 50 ng/ml PMA with 1,000 ng/ml Ionomycin (Sigma Aldrich) or medium alone for 72 h.

### Cytokine Determination

Levels of IFN-γ and IL-17 were measured in culture supernatants by standard sandwich ELISA following the manufacturer's instructions (BD Biosciences). These antibodies were used for coating and detection, respectively: R4-6A2 and XMG1.2 clones for anti-IFN-γ (BD Biosciences), 17CK15A5 and 17B7 clones for anti-IL-17 (eBioscience). The concentrations were expressed in relation to standard curves constructed by assaying serial dilutions of respective murine standard cytokines.

### Proliferation Assay

For proliferation assays, 2.5 × 10^5^ CD4^+^ T cells purified from spleen of untreated OT-II mice were labeled with CFSE dye and co-cultured with 2.5 × 10^5^ LNs cells obtained from immunized mice 6 h after footpad injection. After 72 h of culture with medium alone or 200–400 μg/ml OVA, cells were stained with anti-mouse CD3 (145-2C11), CD4 (RM4-5), Vαβ (MR9-4), and Ki67 (SolA15). The percentage of OT-II CD4^+^ T cell proliferation was determined by CFSE dilution and Ki67 expression by flow cytometry analysis.

### Cell Purification

Neutrophils were isolated from BM and LNs with magnetic microbeads following the manufacturer's instructions (Miltenyi Biotec). A single cell suspension was incubated with MACS anti-Ly6G-biotin plus anti-biotin magnetic microbeads (Miltenyi Biotec). Ly6G^+^ cell purity was > 95% as assessed by flow cytometry.

CD4^+^ T cell purification was performed from a single cell suspension of splenocytes from OT-II mice treated with RBC lysing buffer. Cells were incubated with fluorochrome-labeled anti-CD4 (RM4-5) for 30 min at 4°C, washed with MACS buffer and isolated using cell sorting on a FACSAria II cell sorter (BD Biosciences). The purity of sorted cells was always >98%.

### Neutrophil Depletion *in vivo*

For neutrophil depletion, immunized mice were treated with a monoclonal antibody purified from NIMP-R14 hybridoma, which was provided by Dr. Geneviève Milon and Dr. Salah Mecheri (Institut Pasteur, Paris, France). Total serum IgG from rat was used as isotype control. First, 150 μg of antibody were intraperitoneally administered 24 h before footpad injection, then 75 μg of antibody was injected daily until the animals were sacrificed.

### IC Incubation and Adoptive Transfer

Purified neutrophils from BM of WT or PD-L1 Knock-out (KO) mice were incubated for 1 h at 37°C in a 5% CO_2_ humidified incubator with previously formed OVA/anti-OVA IC (1,25 × 10^6^ cel/ml of IC). The IC were formed by incubation of inactivated polyclonal anti-OVA rabbit sera (dilution 1/75) (NATOCOR) with OVA to a final concentration of 1.6 μg/ml for 30 min at 37°C. Then, 6 × 10^6^ IC-incubated neutrophils or SS were injected into the footpad of WT immunized mice 10 days after OVA/IFA booster immunization and, 36 h later, popliteal LNs were surgically removed and single cell suspensions were prepared for analysis by Flow Cytometry.

### Statistical Analysis

Results are expressed as the mean ± SEM. Data were analyzed using GraphPad Prism software. Data analysis included the unpaired Student *t* test, one-way ANOVA, and two-way ANOVA followed by a Bonferroni *post-hoc* test. All data were considered statistically significant for *p* < 0.05.

## Results

### Transient Influx of OVA^+^ Neutrophils to LNs of OVA/CFA + OVA/IFA Immunized Mice After OVA Footpad Injection

The formation of IC required to induce neutrophil migration to LNs was performed by the following experimental approach. First, C57BL/6 mice received one immunization of OVA/CFA and 15 days later were boosted with OVA/IFA. To evaluate the arrival of neutrophils in LNs, 10 days after the last immunization the mice were injected with OVA-FITC into the hind footpad and draining popliteal lymph nodes (dLNs) were obtained at different time points. As a control, SS footpad injections were made and the popliteal LNs obtained were named non-draining lymph nodes (ndLNs).

**Figure 1:**
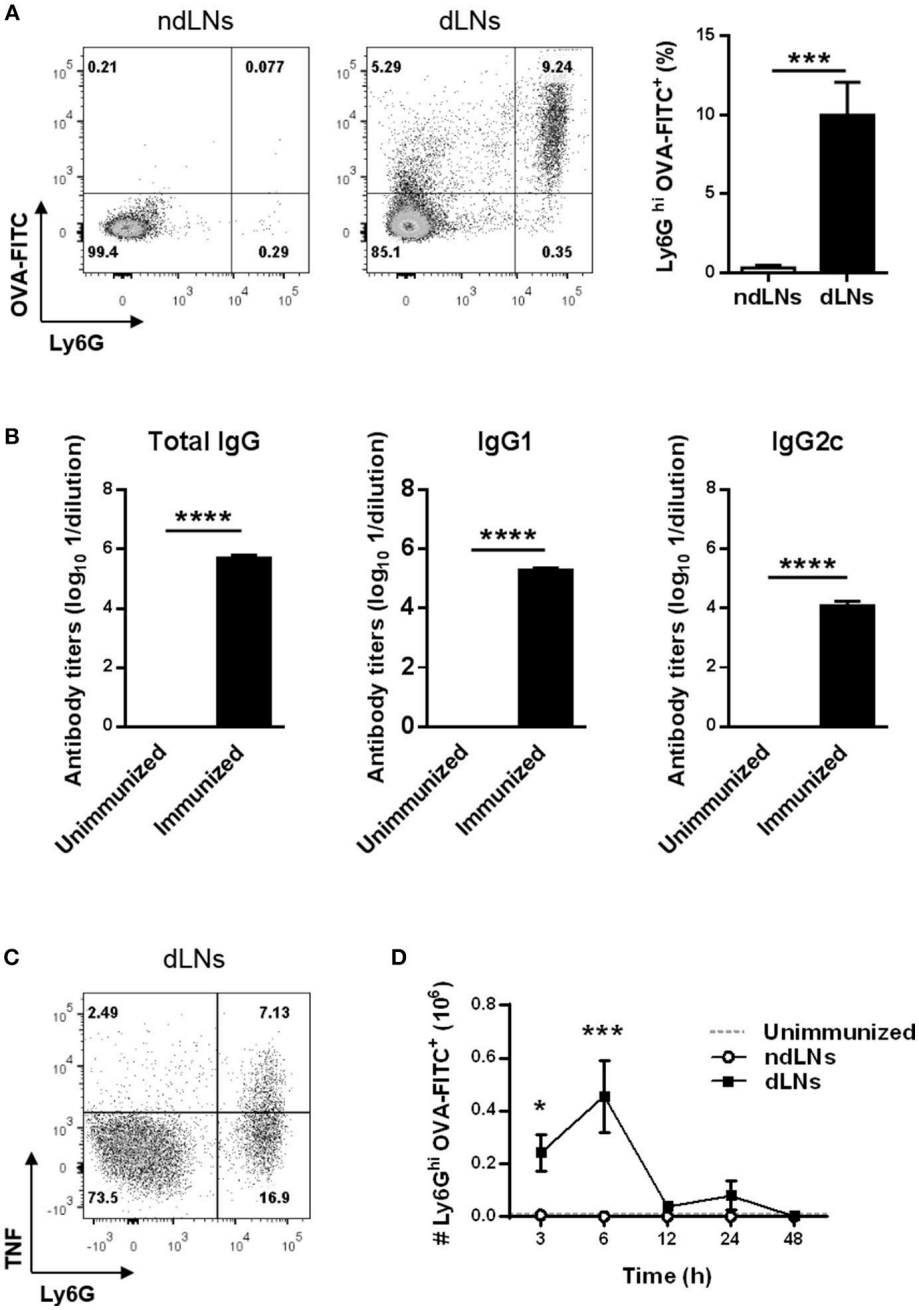
Transient influx of OVA^+^ neutrophils to LNs of OVA/CFA + OVA/IFA immunized mice after OVA footpad injection. C57BL/6 mice were immunized at day 0 with OVA/CFA and at day 15 with OVA/IFA. Ten days after the second immunization, mice were injected in the hind footpad with OVA-FITC or SS as control to obtain dLNs and ndLNs, respectively. **(A)** Flow cytometry analysis of Ly6G^hi^ OVA-FITC^+^ neutrophils in dLNs and ndLNs obtained 6 h after footpad injection. Representative dot plots with numbers indicating percentage of cells and bar graph of the analysis. **(B)** OVA-specific total IgG, IgG1 and IgG2c titers from plasma obtained 10 days after last immunization compared with unimmunized animals. **(C)** Representative dot plot of flow cytometry for intracellular staining of TNF on Ly6G^hi^ alive gated cells. Numbers indicate the percentage of cells. dLNs cells obtained 6 h after OVA footpad injection were cultured *in vitro* without re-stimulation. **(D)** Absolute number of Ly6G^hi^ OVA-FITC^+^ neutrophils in LNs obtained from immunized mice at different time points after footpad injection. In the dotted line, normal values of LNs from unimmunized mice are shown as reference. Results are representative of three independent experiments and are expressed as mean ± SEM (*n* = 4/group); *p < 0.05, ***p < 0.001, ****p < 0.0001.

LN cells from immunized mice were analyzed by flow cytometry to identify OVA^+^ neutrophils by their high expression of the Ly6G marker and the presence of OVA-FITC. As shown in Figure 1A, 6 h after footpad injection, OVA^+^ neutrophils arrived exclusively in dLNs and were absent in ndLNs.

The arrival of OVA^+^ neutrophils in dLNs happened together with OVA-specific antibodies in plasma. We found elevated levels of total IgG, IgG1 and IgG2c OVA-antibody in plasma from immunized mice 10 days after OVA/IFA booster immunization (Figure 1B). Besides, neutrophils in dLNs exhibited a positive cytoplasmic staining for TNF (Figure 1C).

We next studied the kinetics of neutrophil migration to dLNs and evaluated how long these cells remain there. The highest number of OVA^+^ neutrophils in dLNs was detected 6 h after OVA injection and, at 12 h, the number of these cells had decreased, reaching basal levels (Figure 1D). This matches the kinetics of total neutrophils, because the majority of neutrophils were OVA^+^ (Supplementary Figure 1A). These results showed that neutrophil influx to dLNs was rapid, as they were found 3 h after OVA footpad injection, and transient, because at 48 h no more were detected. In ndLNs, the number of neutrophils and OVA^+^ neutrophils was insignificant at all times studied.

Collectively, our data indicate that the injection of OVA into the footpad of OVA/CFA + OVA/IFA-immunized mice that have anti-OVA antibodies induces a transient migration of OVA^+^ neutrophils to dLNs that produce TNF.

### Neutrophil Influx to dLNs Induces CD4^+^ T Cell Expansion

**Figure 2:**
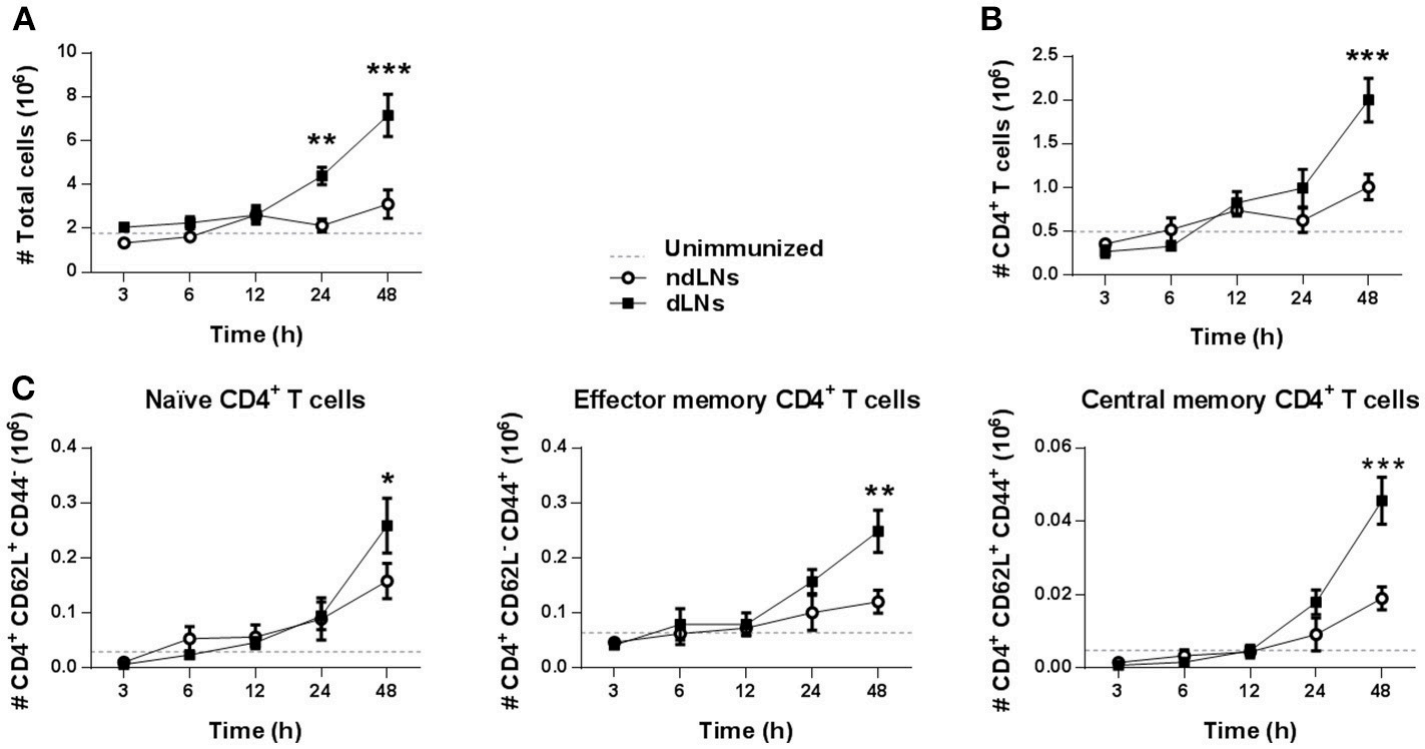
CD4^+^ T cells expansion after neutrophils influx to dLNs. Flow cytometry analysis of cell populations in dLNs and ndLNs obtained from immunized mice at different time points after footpad injection. In the dotted line, normal values of LNs from unimmunized mice are shown as reference. Absolute number of **(A)** total cells; **(B)** CD4^+^ T cells; **(C)** CD62L^+^CD44^−^ naïve, CD62L^−^ CD44^+^ effector memory, and CD62L^+^ CD44^+^ central memory CD4^+^ T cells. Results are representative of three independent experiments and are expressed as mean ± SEM (*n* = 4/group); ns, not significant, *p < 0.05, **p < 0.001, ***p < 0.001.

To study the impact of neutrophil recruitment to dLNs on the other cell populations present there, we first examined the total number of LN cells. As shown in Figure 2A, the total number of cells in dLNs increased but, surprisingly, not when the neutrophils were present but later at 24–48 h, indicating that other cells were increasing in the dLNs after the neutrophil influx.

It has been reported that neutrophils in LNs are able to promote CD4^+^ T cell proliferation (26). We therefore decided to study CD4^+^ T cells in dLNs and observed that, 48 h after OVA footpad injection, their number increased, which matches the time of the total increase of dLN cells (Figure 2B). We also evaluated CD4^+^ T cell subsets and found that, at 48 h, there was an increase of naïve, effector memory, and central memory CD4^+^ T cells in dLNs, indicating a massive increase of this population (Figure 2C).

In addition, 24 h after OVA injection, we observed an increase of CD11c^+^ DCs in dLNs. However, when we analyzed OVA^+^ DCs, the highest number of OVA^+^CD11c^+^ cells was observed 6 h after OVA footpad injection in dLNs (Supplementary Figure 1B). As the number of OVA^+^ CD11c^+^ cells is at least ten times less than that of OVA^+^ Ly6G^hi^ cells, it should be noted that the main antigen carrier cells were neutrophils.

**Figure 3:**
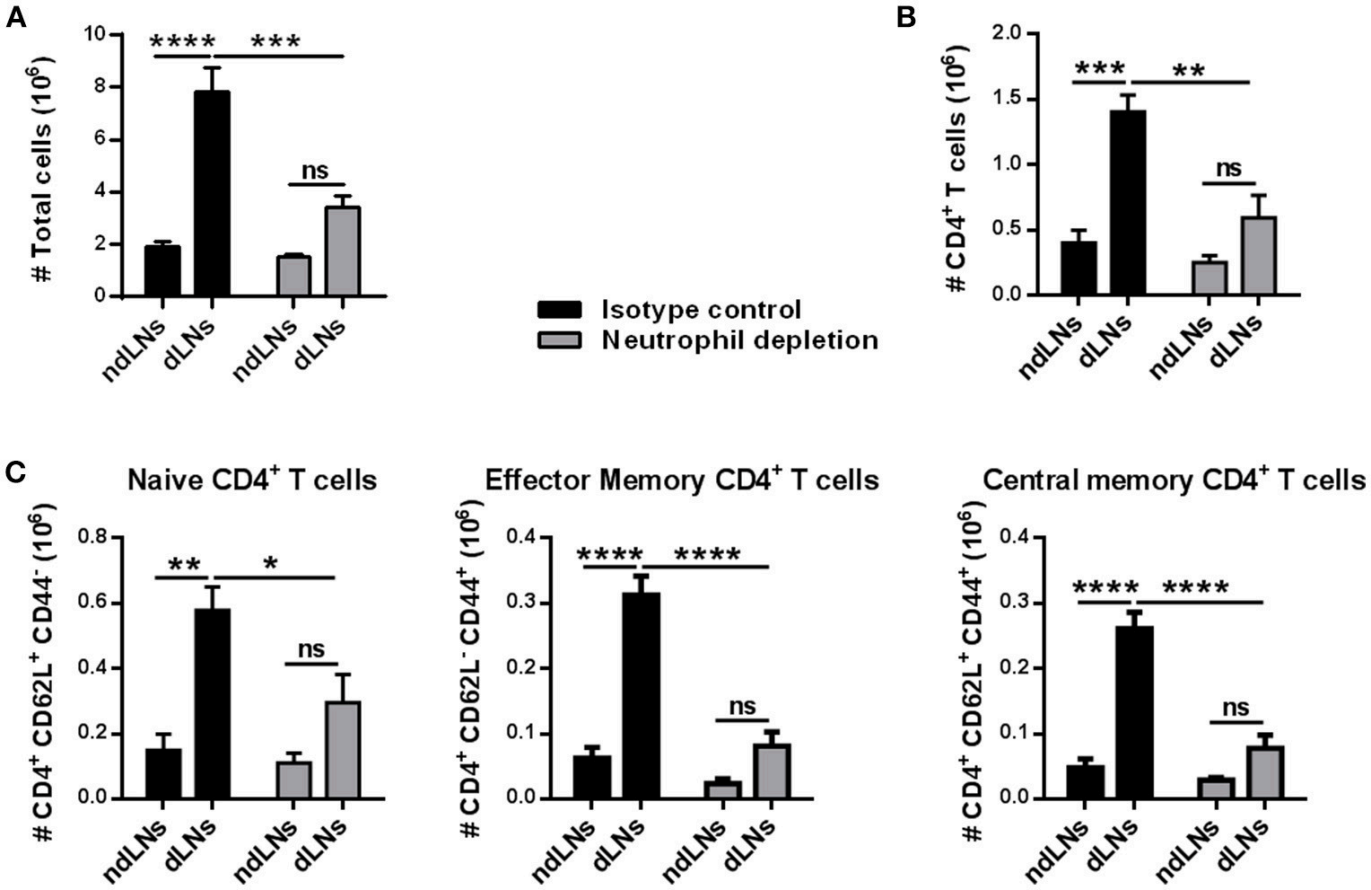
The increase of CD4^+^ T cells depends on the arrival of neutrophils to the LNs. Immunized mice were treated with isotype control or NIM-R14 antibody to deplete neutrophils; dLNs and ndLNs were obtained 56 h after OVA or SS footpad injection. Absolute number of **(A)** total cells; **(B)** CD4^+^ T cells; **(C)** CD62L^+^CD44^−^ naïve, CD62L^−^ CD44^+^ effector memory and CD62L^+^ CD44^+^ central memory CD4^+^ T cells were determined by flow cytometry. Results are representative of three independent experiments and are expressed as mean ± SEM (*n* = 4–5/group); ns, not significant, *p < 0.05, **p < 0.001, ***p < 0.001, ****p < 0.0001.

To evaluate whether the increase of CD4^+^ T cells observed in dLNs was due to neutrophil arrival, immunized mice were treated with NIMP-R14 antibody to deplete neutrophils *in vivo*. The experimental approach used and the neutrophil depletion controls are shown in (Supplementary Figures 2A–C). At 56 h after OVA footpad injection, in neutrophil-depleted mice we did not observe a significant increase of LN total cells or of CD4^+^ T cells in dLNs, unlike the animals that received the isotype control antibody (Figures 3A,B). Similar results were observed when we analyzed naïve, effector memory and central memory CD4^+^ T cell subsets (Figure 3C). Thus, the influx of neutrophils induced the increase of CD4^+^ T cell populations observed in dLNs from immunized mice after OVA footpad injection.

These findings indicate that the presence of OVA^+^ neutrophils in dLNs influences the CD4^+^ T cell population. Neutrophil influx was necessary for CD4^+^ T cell expansion in dLNs.

### CD4^+^ T Cell Activation and Proliferation Is Induced in dLNs After OVA^+^ Neutrophil Influx

As the number of CD4^+^ T cells increased in dLNs, we next studied whether the presence of OVA^+^ neutrophils in LNs subsequently affected the CD4^+^ T cell response in dLNs obtained 24 h after OVA footpad injection.

**Figure 4:**
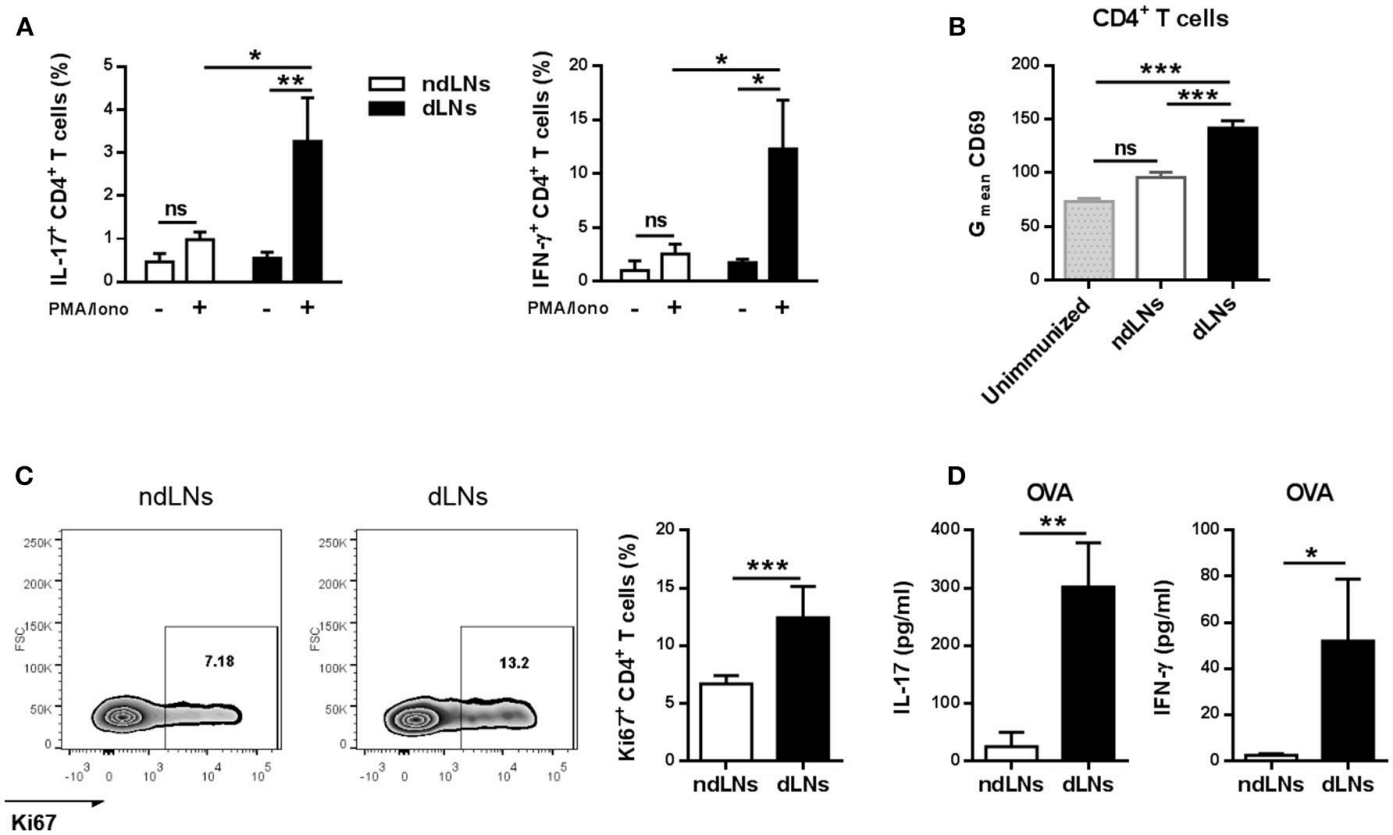
CD4^+^ T cell activation and proliferation is induced in dLNs after OVA^+^ neutrophil influx. dLNs and ndLNs cells were obtained from immunized mice 24 h after OVA or SS footpad injection. **(A)** Cells were cultured for 72 h with PMA and Ionomycin or medium alone and later the percentage of IL-17^+^ CD4^+^ T cells and IFN-γ^+^ CD4^+^ T cells was determined by flow cytometry analysis. **(B)** Geometric mean (G_mean_) of CD69 levels in CD4^+^ T cells. Unimmunized mice were used as control. **(C)** Representative dot plot of Ki67^+^ CD4^+^ T cells with numbers indicating percentage of gated cells and bar graph of the analysis. **(D)** Cells were cultured for 72 h with OVA (100 μg/mL) or medium alone to measure IL-17 and IFN-γ in the culture supernatant by ELISA; data represent stimulated minus unstimulated levels of cytokines. Results are representative of three independent experiments and are expressed as mean ± SEM (*n* = 4–5/group); ns, not significant, *p < 0.05, **p < 0.001, ***p < 0.001.

First, we examined the cytokines produced by CD4^+^ T cells by flow cytometry analysis of LN cells stimulated *in vitro*, and found that the percentages of IL-17^+^ CD4^+^ T cells and IFN-γ^+^ CD4^+^ T cells were significantly higher in dLNs than in ndLNs (Figure 4A). No double positive IL-17^+^ IFN-γ^+^ CD4^+^ T cells were detected (Supplementary Figure 3).

To evaluate whether CD4^+^ T cells from dLNs were activated, we analyzed the expression of the cell surface marker CD69. CD4^+^ T cells from dLNs had higher levels of CD69 than those present in ndLNs, the levels of which were similar to those of an unimmunized mouse (Figure 4B).

Then we wondered if the CD4^+^ T cells that were more activated in dLNs were also proliferating, and therefore we performed flow cytometry analysis of Ki67 levels. CD4^+^ T cells from dLNs showed a higher percentage of Ki67 levels, indicating augmented proliferative response (Figure 4C).

Finally, to evaluate if the increase of CD4^+^ T cell cytokine production was antigen-specific, LN cells were stimulated *in vitro* with OVA for 72 h. As shown in Figure 4D, we found increased IL-17 and IFN-γ levels in the culture supernatant from dLNs, while IL-5 and IL-4 concentrations were not detected (data not shown).

These results demonstrate that, after the migration wave of OVA^+^ neutrophils in dLNs, CD4^+^ T cells proliferate and also upregulate CD69 and produce IL-17 and IFN-γ, which indicates that they are more activated.

### Neutrophils in dLNs Enhance CD4^+^ T Cell Proliferation

**Figure 5:**
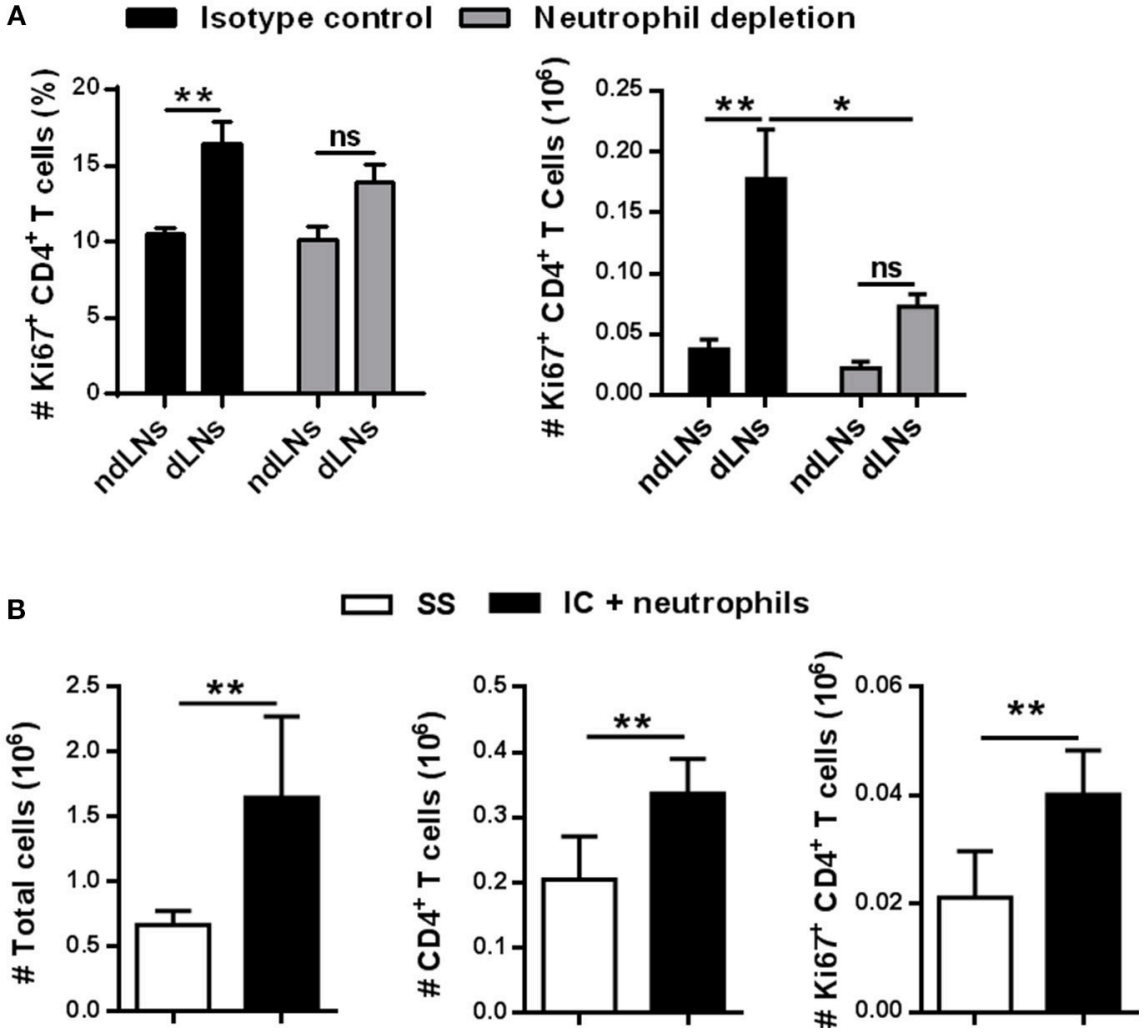
IC-incubated neutrophils enhance CD4^+^ T cell proliferation. **(A)** Percentage and absolute number of Ki67^+^ CD4^+^ T cells in dLNs and ndLNs obtained 56 h after OVA or SS footpad injection of immunized mice treated with isotype control or NIMP-R14 antibody to deplete neutrophils. **(B)** Neutrophils were purified from BM and incubated *in vitro* with IC for adoptive transfer experiments. IC-incubated neutrophils or SS were transferred into the footpad of immunized mice 10 days after OVA/IFA booster immunization; 36 h later the absolute number of LN total cells, CD4^+^ T cells, and Ki67^+^ CD4^+^ T cells was analyzed by flow cytometry. Results are representative of three independent experiments and are expressed as mean ± SEM (*n* = 4–5/group); ns, not significant, *p < 0.05, **p < 0.001.

Considering that an increased number of proliferating CD4^+^ T cells was observed in dLNs, we decided to evaluate whether the influx of neutrophils impacts CD4^+^ T cell proliferation. We performed flow cytometry analysis of neutrophil-depleted mice to study proliferating Ki67^+^ CD4^+^ T cells in LNs 56 h after OVA footpad injection. As shown in Figure 5A, neutrophil-depleted mice did not have a significant increase of percentage or absolute number of Ki67^+^ CD4^+^ T cells in dLNs compared to ndLNs.

To corroborate that neutrophils which migrate to dLNs were required to increase CD4^+^ T cell proliferation, adoptive transfer experiments were performed. First, neutrophils purified from BM were incubated *in vitro* with previously formed IC. As control, we observed that neutrophils upregulated their CD11b levels after IC incubation (Supplementary Figure 4). Then, these neutrophils were transferred to the footpads of immunized mice 10 days after OVA/IFA booster immunization, instead of the OVA footpad injection. In our previous work (22), we demonstrated that, with this experimental strategy, neutrophils that were incubated with IC reach dLNs mimicking what happens when IC is formed *in vivo*. As shown in Figure 5B, the number of total cells, CD4^+^ T cells, and proliferative Ki67^+^ CD4^+^ T cells increased more in LNs obtained from mice that were transferred with IC-incubated neutrophils compared with LNs from mice that received the SS injection.

These results show that neutrophils that reach dLNs after IC incubation are essential to induce CD4^+^ T cell proliferation in dLNs.

### CD4^+^ T Cell Proliferation Is Restricted When Neutrophils Are Present in dLNs

**Figure 6:**
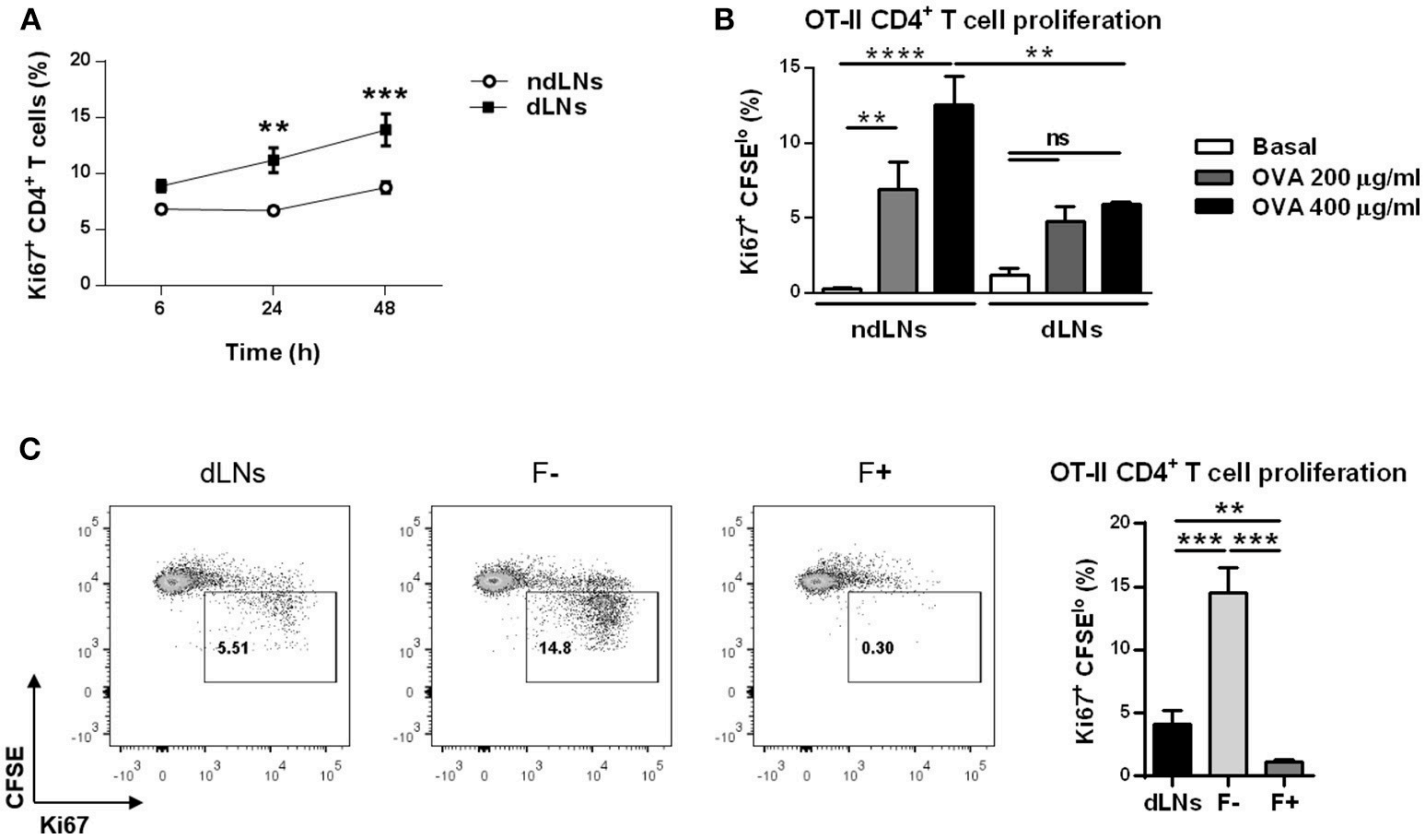
CD4^+^ T cell proliferation is restricted when neutrophils are present in dLNs. **(A)** Percentage of Ki67^+^ CD4^+^ T cells determined by flow cytometry analysis of dLNs and ndLNs cells obtained at 6, 24, and 48 h after immunized mice received OVA or SS footpad injection. **(B,C)** *in vitro* proliferation assay of CD4^+^ T cells isolated from untreated OT-II mice and labeled with CFSE dye. These cells were co-cultured with the following cells obtained from immunized mice 6 h after footpad injection: **(B)** dLNs or ndLNs; **(C)** dLNs, negative fraction (F-) that consists of dLNs without neutrophils or positive fraction (F+) of neutrophils purified from dLNs. After 72 h culture with medium alone, 200 or 400 μg/ml OVA, cells were stained with anti-CD3, anti-CD4, anti-Vαβ, anti-Ki67, and percentage of OT-II CD4^+^ T cell proliferation was determined by CFSE dilution and Ki67 expression. In **(C)** representative dot plots are also shown, with numbers indicating percentage of proliferating gated cells and graph bars representing 400 μg/ml OVA minus basal levels. Results are representative of three independent experiments and are expressed as mean ± SEM (*n* = 4/group); ns, not significant, **p < 0.001, ***p < 0.001, ****p < 0.0001.

The results above showed that CD4^+^ T cell proliferation was enhanced in dLNs where IC-incubated neutrophils had previously arrived. However, as shown in Figure 6A, when we studied CD4^+^ T cell proliferation at different time points after footpad injection, we observed that at 6 h there was no significant difference of percentages of Ki67^+^ CD4^+^ T cells between ndLNs and dLNs. In contrast, at longer time points there were higher levels of Ki67^+^ CD4^+^ T cells in dLNs. Taking into account that the higher number of OVA^+^ neutrophils in dLNs was observed at 6 h after OVA footpad injection and that they were no longer present from 12 h onwards, we decided to study whether neutrophils were restricting CD4^+^ T cell proliferation.

Next, we decided to evaluate whether the neutrophils present in dLNs obtained at 6 h after OVA footpad injection could modulate OVA-specific naïve CD4^+^ T cells proliferative response. We performed *in vitro* proliferation assays using CFSE-labeled OVA-specific CD4^+^ T cells obtained from untreated OT-II mice stimulated with OVA. These cells were co-cultured with LN cells from WT immunized mice obtained at 6 h after footpad injection, to evaluate whether the presence of neutrophils in those co-cultures inhibited OT-II CD4^+^ T cell proliferation. After 72 h of culture, we observed that the proliferative response was lower when OT-II CD4^+^ T cells were cultivated with dLNs than in cells cultured with ndLNs cells (Figure 6B). This may indicate that neutrophils present in dLNs were restricting CD4^+^ T cell proliferation.

To confirm this, we next worked with dLN cells obtained from immunized mice at 6 h after OVA footpad injection that were depleted of neutrophils with magnetic beads (F-). As controls, we also used total dLN cells and the neutrophils purified from these (F+). These cells were co-cultured with CFSE-labeled OT-II CD4^+^ T cells and stimulated *in vitro* with OVA. As expected, in the co-culture in which neutrophils were absent, the OT-II CD4^+^ T cell proliferative response was greater than in the co-cultures with total dLN cells (Figure 6C). This indicated that neutrophils present in dLNs were suppressing CD4^+^ T cell proliferation. Although the lowest percentage of OT-II CD4^+^ T cell proliferation was found when co-cultured with neutrophils alone, this may be due not only to the inhibitory effect of neutrophils but might also be attributed to the lack of APCs required to mount a CD4^+^ T cell response.

These findings demonstrate that CD4^+^ T cell proliferation is restricted when neutrophils are present in dLNs.

### PD-L1^+^ Neutrophils in dLNs Impair CD4^+^ T Cells Proliferation

**Figure 7:**
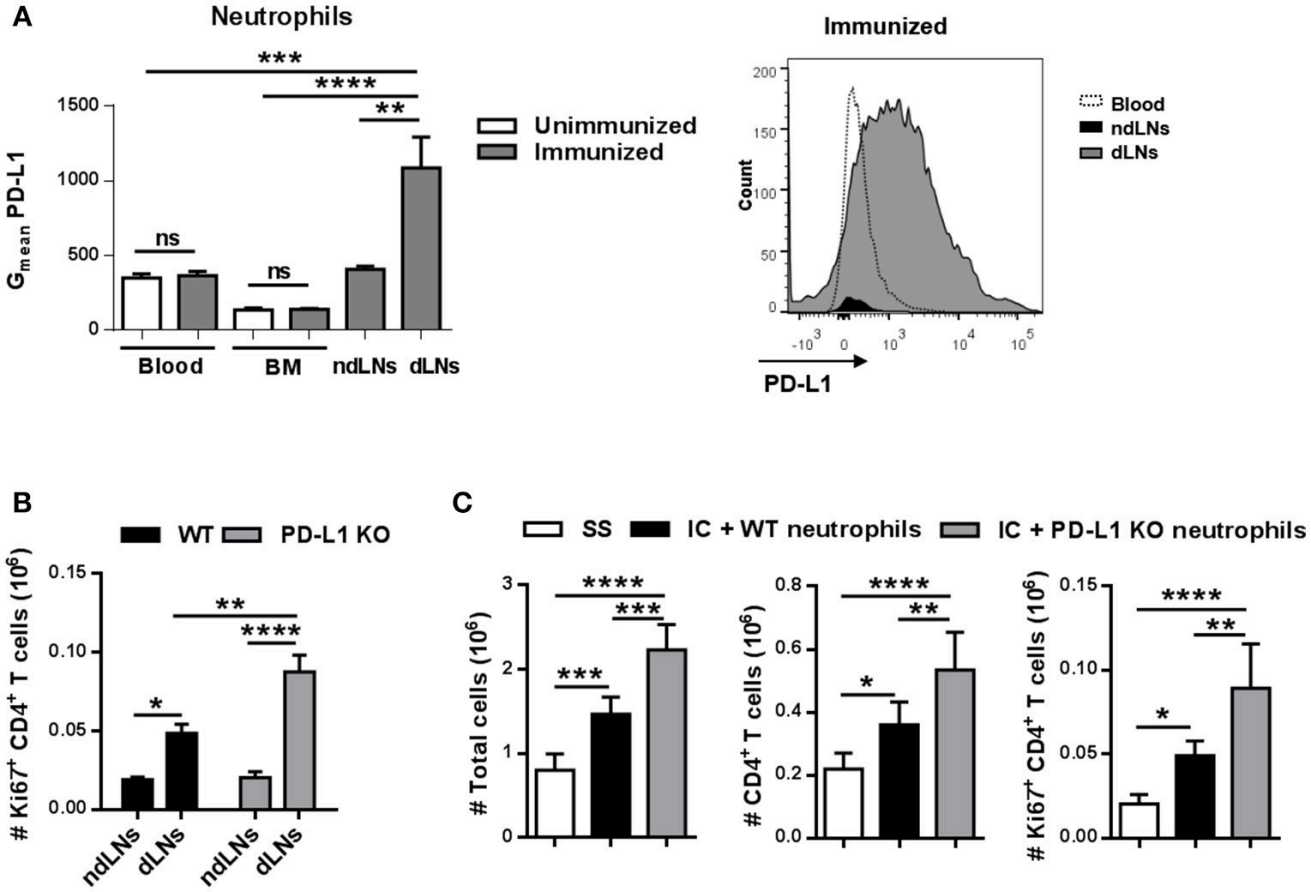
PD-L1^+^ neutrophils in dLNs impair CD4^+^ T cell proliferation. **(A)** Geometric mean (G_mean_) of PD-L1 levels on Ly6G^hi^ neutrophils present in dLNs, ndLNs, blood, and BM obtained from immunized mice 6 h after footpad injection; blood and BM cells from unimmunized mice were used as reference. Representative histogram of PD-L1 levels on neutrophils from blood, dLNs, and ndLNs of immunized mice is also shown. **(B)** Absolute number of Ki67^+^ CD4^+^ T cells in dLNs and ndLNs obtained from WT or PD-L1 KO immunized mice at 48 h after footpad injection. **(C)** For adoptive transfer experiments, neutrophils were purified from BM of WT or PD-L1KO untreated mice and incubated *in vitro* with IC. IC-incubated neutrophils from WT or PD-L1KO mice or saline solution (SS) were transferred into the footpad of WT immunized mice 10 days after OVA/IFA booster immunization; 36 h later, the absolute number of LN total cells, CD4^+^ T cells, and Ki67^+^ CD4^+^ T cells was analyzed by flow cytometry. Results are representative of three independent experiments and are expressed as mean ± SEM (*n* = 4/group); ns, not significant, *p < 0.05, **p < 0.001, ***p < 0.001, ****p < 0.0001.

PD-L1 is a transmembrane protein that binds to the receptor PD-1 present in lymphocytes and transmits an inhibitory signal that, among other functions, reduces the proliferation of T cells (27). We next analyzed PD-L1 levels in neutrophils and observed that the neutrophils present in dLNs had higher levels of PD-L1 than the few of them found in ndLNs obtained from immunized mice 6 h after footpad injection (Figure 7A). Neutrophils from BM and blood from immunized mice and from unimmunized mice were used as controls, demonstrating that only neutrophils that reached dLNs upregulate PD-L1.

To evaluate whether PD-L1^+^ neutrophils were restricting the CD4^+^ T cell response *in vivo*, PD-L1 KO mice were immunized and, 48 h after OVA footpad injection, we observed a higher number of Ki67^+^ CD4^+^ T cells in dLNs than in WT immunized mice (Figure 7B). We also performed adoptive transfer experiments in which *in vitro* IC-incubated neutrophils isolated from BM of WT or PD-L1 KO untreated mice were transferred to the footpad of WT immunized mice. As shown in Figure 7C, 36 h after adoptive transfer, the animals that received PD-L1 KO neutrophils showed a higher number of total LN cells, CD4^+^ T cells, and proliferative Ki67^+^ CD4^+^ T cells than those that were transferred with WT neutrophils, whose numbers were already higher than those that received SS. Thus, IC-incubated neutrophil migration to LNs induces CD4^+^ T cell proliferation and, at the same time, this proliferation is restricted by PD-L1.

Collectively, this data strongly supports a dual role for neutrophils that reach dLNs in the modulation of CD4^+^ T cell response. After the influx of neutrophils to LNs dependent on IC formation, CD4^+^ T cells are more activated and proliferating but also the expansion of CD4^+^ T cells is restricted by the PD-L1 molecule expressed by the neutrophils.

## Discussion

Neutrophils are recognized for performing a critical role in pathogen clearance, inflammation, and wound healing (2) but they also have the ability to interact and regulate other cells of the immune system (4, 5, 28). Additionally, it has been reported that there are different stimuli, like infection with bacteria (26, 29), protozoan parasites (30, 31), viruses (32, 33), as well as the tumor microenvironment (34), that generate neutrophil influx to the LN, while under normal physiological conditions these cells are absent in this organ. Our group was the first to demonstrate that the formation of IC is a stimulus capable of inducing the migration of neutrophils to LNs under inflammatory conditions without ongoing infection (21).

The study of the effects of IC on neutrophils has great relevance since IC-induced activation plays a central role in the pathogenesis of some autoimmune inflammatory diseases (35). It is well known that the synovial fluid of patients with active Rheumatoid Arthritis contains large quantities of IC that can activate infiltrating neutrophils (36). However, the role of neutrophils that migrate to LNs in this scenario is little known.

Previously, we showed that immunized mice that have specific antibodies, when challenged with the same antigen, generate the IC formation necessary for the migration of neutrophils to LNs (21, 22). In those studies, mice had received 3 immunizations of OVA/CFA and then were challenged with OVA in their footpad. In the present work we changed our immunization protocol to reduce undesirable side effects of CFA that produce skin ulceration (37, 38). The influx of OVA^+^ neutrophils to LNs was induced even when we decreased the number of immunizations and exchanged the CFA adjuvant for IFA in the second immunization; IFA is the incomplete Freund's adjuvant form that lacks the mycobacterial components. Indeed, the specific antibody production measured in this work was consistent with the previous immunization model. Moreover, neutrophil trafficking into LNs also occurred when neutrophils were incubated with IC *in vitro* and then transferred to the animal's footpad.

Neutrophils are known to provide the first line of defense by being quickly recruited at a site of injury, where they fulfill their protection function and die within a few hours (2, 39). However, it has been reported that some stimuli may increase the lifespan of neutrophils (40). For this reason, we studied the kinetics of neutrophils migration to dLNs and evaluated how long these cells remain there. We observed that neutrophil influx to dLNs was rapid, as they were found 3 h after OVA footpad injection, and the highest number of OVA^+^ neutrophils in dLNs was detected at 6 h. Also, neutrophil influx to dLNs was transient, since 12 h after OVA footpad injection the number of these cells had decreased reaching basal levels and at 48 h no more were detected.

Many functions have been attributed to neutrophils that reach the LNs, depending on the stimulus that induced the migration, the microenvironment of the LNs, and the timing (20). Previously, we observed by confocal microscopy that there were neutrophils in the T-cell zone of dLNs 6 h after OVA footpad injection (22), suggesting that there might be some crosstalk between neutrophils and T lymphocytes. In this work, we have shown that the number of CD4^+^ T cells increased in dLNs after the influx of OVA^+^ neutrophils. We observed that the expansion of CD4^+^ T cells is not exclusive to any particular subset, but rather a massive expansion of this population occurred, since there was an increase of naïve and effector memory CD4^+^ T cells as well as of central memory CD4^+^ T cells responsible for long-term protection. Contrary to our results, Hor et al. showed that CD4^+^ T cell expansion and their levels of CD69, CD62L, and CD44 molecules were unaffected after neutrophil influx to LNs, in a mouse model of cutaneous herpes simplex virus type 1 (33). This suggests that the ability of neutrophils to induce CD4^+^ T cell expansion may be context-dependent, and is likely to be determined by the type of stimulus that induces neutrophil migration to LNs.

In agreement with other authors (14, 31, 41), we found that neutrophils were able to modulate adaptive immunity by regulating T cell polarization. The CD4^+^ T cells obtained from dLNs after the influx of neutrophils were more activated and produced higher levels of IFN-γ and IL-17 cytokines than those from ndLNs.

We also demonstrated that neutrophil migration to dLNs enhances CD4^+^ T cell proliferation 24 to 56 h after OVA footpad injection. The increase of Ki67^+^ CD4^+^ T cells depends on the arrival of neutrophils, since neutrophil-depleted mice showed lower levels of proliferation. The adoptive transfer of *in vitro* IC-incubated neutrophils also induced an increase of Ki67^+^ CD4^+^ T cells in LNs. In agreement with our results, it has been reported that neutrophils promote CD4^+^ T cell proliferation in LNs after immunization with killed *Staphylococcus aureus* (26). The expansion of CD4^+^ T cells observed in dLNs is a consequence, at least in a large extent, of CD4^+^ T cells proliferation, although we cannot discard that the recruitment of these cells could also be involved.

The neutrophils that reach dLNs in our experimental model could be contributing to the activation and expansion of CD4^+^ T cells through different mechanisms. We demonstrated that neutrophils perform the antigen (OVA) uptake and transport from the footpad to the dLNs, where they reach antigen-specific lymphocytes generated by immunization, as has been reported in other studies in which neutrophils shuttle *S. aureus* particles (26) and live bacilli of BCG (29) from skin to LNs.

It has also been shown that neutrophils could directly or indirectly collaborate with antigen presentation to CD4^+^ T cells. Several studies have reported that neutrophils can behave as APCs (12, 42, 43), and indeed Hülsdünker et al. recently reported that, at the onset of a graft-vs.-host disease, neutrophils migrate to mesenteric LNs, and have the ability to present a peptide in the context of MHC-II inducing CD4^+^ T cell proliferation (44). Unlike this and other reports (26, 44), we did not observe elevated levels of MHC-II or CD86 in neutrophils that reached dLNs (data not shown). Actually, in our proliferative assay of OT-II CD4^+^ T cells co-cultivated with neutrophils purified from dLNs, we observed that the proliferation of CD4^+^ T cells was abolished. We consider that this result is due to the combination of suppression exerted by the neutrophil and a lack of professional APCs in the culture. Vono et al. reported that human neutrophils can function as APCs to memory CD4^+^ T cells, although in the expression of co-stimulatory molecules as well as in the lymphocyte proliferative assay, neutrophils were less efficient compared to professional APCs (13).

Alternatively, neutrophils may influence CD4^+^ T cell responses indirectly by acting upon DCs (45). We previously demonstrated that neutrophils die in dLNs by apoptosis (21) and therefore a possible explanation of why we observed OVA^+^ DCs in dLNs could be that neutrophils had died in dLNs, and their cellular debris including OVA may be phagocytosed by DCs and they then present the antigen to CD4^+^ T cells. However, further experiments are needed to confirm this hypothesis. We cannot exclude the possibility that DCs can uptake the antigen in the footpad and then migrate to dLNs, nor that soluble OVA reaches the LNs and then resident DCs uptake it. Although these last two options could be contributing to the activation and proliferation of CD4^+^ T cells, we cannot underestimate the importance of neutrophil influx to dLNs because the increase of Ki67^+^ CD4^+^ T cells was impaired in neutrophil depletion experiments.

Finally, we showed that neutrophils that reached dLNs are TNF producers. It has been reported that TNF is a pleiotropic cytokine synthesized by several cell types including activated neutrophils and can cause the activation and expansion of CD4^+^ T cells (46). Therefore, the TNF produced by neutrophils in dLNs could be contributing to the CD4^+^ T cell response observed.

Although we suggest that the ability of the neutrophil to transport the antigen to LNs, to produce TNF and maybe collaborate directly or indirectly to antigen presentation, may cause the activation and expansion of CD4^+^ T cells, we do not discard the possibility that other mechanisms may also be contributing to these actions.

On the other hand, many reports have established that, in certain contexts, neutrophils acquire inhibitory functions and suppress CD4^+^ T cell (14, 15, 47). In our *in vitro* proliferation assays, we observed that the presence of neutrophils restricts CD4^+^ T cell antigen-specific proliferation. In this work, we describe for the first time that neutrophils that reach dLNs enhance the activation and expansion of CD4^+^ T cells and at the same time restrict CD4^+^ T cell proliferation, probably as a compensatory mechanism to avoid harmful excessive response.

Several mechanisms used by neutrophils for T cell inhibition have been reported, including soluble mediators, and cell-to-cell contact (5). We assessed the ability of neutrophils from dLNs to produce nitric oxide as a suppressor mechanism and their levels were undetectable (data not shown). Also, we evaluated if those neutrophils were producing IL-10 and, although we observed a small percentage of IL-10^+^ neutrophils by flow cytometry, there were no significant differences in IL-10 levels of dLNs and ndLNs culture supernatant, suggesting that this cytokine is not implicated in neutrophil suppressive capacity (data not shown).

Interestingly, we found that only neutrophils that reach dLNs upregulate PD-L1, a ligand that has been described as causing the inhibition of CD4^+^ T cell proliferation (27). It has been reported in human (17) and murine (19) endotoxemia that IFN-γ-stimulated neutrophils acquired the capacity to suppress lymphocyte proliferation through the expression of PD-L1. In our model, 48 h after footpad injection with OVA, immunized PD-L1KO mice showed a higher number of Ki67^+^ CD4^+^ T cells than WT immunized mice. In addition, the adoptive transfer of IC-incubated PD-L1 KO neutrophils enhanced the proliferation of CD4^+^ T cells even more than WT transferred neutrophils, suggesting that PD-L1 expression on neutrophils could be preventing an excessive CD4^+^ T cell proliferation.

Two general features of our *in vivo* model are the generation of an inflammatory state and the formation of IC. In this scenario, neutrophils migrate to dLNs and promote the activation and proliferation of CD4^+^ T cells. At the same time, neutrophils upregulate PD-L1 to avoid excessive CD4^+^ T cell proliferation, resulting in a negative feedback mechanism of CD4^+^ T cell response. It would be useful to learn more about the signals that upregulate PD-L1 in neutrophils, to be able to use that information for the treatment of clinical diseases. In pathologies where a large neutrophil infiltrate is produced, it is important not only to know the pro-inflammatory functions that the neutrophil may exert, but also to consider it as a cell with the capacity to modulate the adaptive immune response.

PD-L1^+^ neutrophils may exert different roles according to the pathology to which they are associated. The presence of these cells in tumor bearing hosts has been reported to be detrimental, since they prevent the tumor cells from being attacked by the adaptive immunity (48). However, in autoimmune diseases which are characterized by a great inflammatory state and the presence of autoantibodies, the expression of PD-L1 by neutrophils might be an important mechanism for keeping a balance between effective immunity, tolerance and immunopathology. In line with this, it has been reported that the frequency of PD-L1-expressing neutrophils were elevated in patients with systemic lupus erythematosus (49) and rheumatoid arthritis (50) in correlation with increased autoimmune antibodies, inflammatory markers, and severity of the disease. In these systemic autoimmune diseases, the expression of PD-L1 by neutrophils may function as a negative feedback mechanism, preventing potential tissue damage caused by excessive autoimmune responses.

It has been also reported in a model of Sepsis-induced Immunosuppression an up-regulation of PD-L1 on neutrophils (51). The correlation of these cells with the severity of the disease suggests that PD-L1^+^ neutrophil levels might be a potential diagnostic biomarker for severe inflammatory diseases.

In conclusion, our study reveals a dual function of neutrophils present in dLNs in terms of CD4^+^ T cell modulation. The arrival of neutrophils into dLNs enhances the activation and proliferation of CD4^+^ T cells, but these cells also impair CD4^+^ T cell proliferation by a PD-L1 dependent mechanism. In our model, neutrophils seem to be exerting a critical role in the maintenance of the homeostatic balance between the activation of lymphocytes in an adaptive immune response and their regulation in order to avoid exacerbated response.

## Author Contributions

SC and MP-P designed the experiments. SC performed the experiments, analyzed data, prepared Figures, and wrote the manuscript. MH collaborated with conducting the experiments and analyzing results. MH, GM, and BM contributed to study design and corrected the manuscript. MP-P conceived and supervised the study and collaborated in manuscript writing. All authors read and approved the final manuscript.

### Conflict of Interest Statement

The authors declare that the research was conducted in the absence of any commercial or financial relationships that could be construed as a potential conflict of interest.

## Abbreviations

DCs: dendritic cells
APCs: antigen presenting cells
PD-L1: programmed death ligand 1
LNs: lymph nodes
OVA: ovalbumin
CFA: complete freund's adjuvant
IC: immune complexes
WT: wild-type
IFA: incomplete freund's adjuvant
SS: saline solution
BM: bone marrow
KO: knock out
dLNs: draining popliteal lymph nodes
ndLNs: non-draining lymph nodes.

## References

[1] Borregaard N. Neutrophils, from marrow to microbes. Immunity (2010) 33:657–70. 10.1016/j.immuni.2010.11.011 21094463

[2] Amulic B Cazalet C Hayes GL Metzler KD Zychlinsky A. Neutrophil function: from mechanisms to disease. Ann Rev Immunol. (2012) 30:459–89. 10.1146/annurev-immunol-020711-074942 22224774

[3] Nicolas-Avila JA Adrover JM Hidalgo A. Neutrophils in homeostasis, immunity, and cancer. Immunity (2017) 46:15–28. 10.1016/j.immuni.2016.12.012 28099862

[4] Mantovani A Cassatella MA Costantini C Jaillon S. Neutrophils in the activation and regulation of innate and adaptive immunity. Nat Rev Immunol. (2011) 11:519–31. 10.1038/nri3024 21785456

[5] Leliefeld PH Koenderman L Pillay J. How neutrophils shape adaptive immune responses. Front Immunol. (2015) 6:471. 10.3389/fimmu.2015.00471 26441976 PMC4568410

[6] Parsa R Lund H Georgoudaki AM Zhang XM Ortlieb Guerreiro-Cacais A Grommisch D. BAFF-secreting neutrophils drive plasma cell responses during emergency granulopoiesis. J Exp Med. (2016) 213:1537–53. 10.1084/jem.20150577 27432941 PMC4986521

[7] Puga I Cols M Barra CM He B Cassis L Gentile M. B cell-helper neutrophils stimulate the diversification and production of immunoglobulin in the marginal zone of the spleen. Nat Immunol. (2011) 13:170–80. 10.1038/ni.2194 22197976 PMC3262910

[8] Kalyan S Kabelitz D. When neutrophils meet T cells: beginnings of a tumultuous relationship with underappreciated potential. Eur J Immunol. (2014) 44:627–33. 10.1002/eji.201344195 24435886

[9] Yang CW Unanue ER. Neutrophils control the magnitude and spread of the immune response in a thromboxane A2-mediated process. J Exp Med. (2013) 210:375–87. 10.1084/jem.20122183 23337807 PMC3570104

[10] Bennouna S Bliss SK Curiel TJ Denkers EY. Cross-talk in the innate immune system: neutrophils instruct recruitment and activation of dendritic cells during microbial infection. J. Immunol. (2003) 171:6052–8. 10.4049/jimmunol.171.11.6052 14634118

[11] van Gisbergen KP Sanchez-Hernandez M Geijtenbeek TB van Kooyk Y. Neutrophils mediate immune modulation of dendritic cells through glycosylation-dependent interactions between Mac-1 and DC-SIGN. J Exp Med. (2005) 201:1281–92. 10.1084/jem.20041276 15837813 PMC2213143

[12] Abi Abdallah DS Egan CE Butcher BA Denkers EY. Mouse neutrophils are professional antigen-presenting cells programmed to instruct Th1 and Th17 T-cell differentiation. Int. Immunol. (2011) 23:317–26. 10.1093/intimm/dxr007 21422151 PMC3082529

[13] Vono M Lin A Norrby-Teglund A Koup RA Liang F Lore K. Neutrophils acquire the capacity for antigen presentation to memory CD4(+) T cells *in vitro* and *ex vivo*. Blood (2017) 129:1991–2001. 10.1182/blood-2016-10-744441 28143882 PMC5383872

[14] Doz E Lombard R Carreras F Buzoni-Gatel D Winter N. Mycobacteria-infected dendritic cells attract neutrophils that produce IL-10 and specifically shut down Th17 CD4 T cells through their IL-10 receptor. J Immunol. (2013) 191:3818–26. 10.4049/jimmunol.1300527 23997221

[15] Tosello Boari J Amezcua Vesely MC Bermejo DA Ramello MC Montes CL Cejas H. IL-17RA signaling reduces inflammation and mortality during Trypanosoma cruzi infection by recruiting suppressive IL-10-producing neutrophils. PLoS Pathogens (2012) 8:e1002658. 10.1371/journal.ppat.1002658 22577359 PMC3343119

[16] Odobasic D Kitching AR Yang Y O'Sullivan KM Muljadi RC Edgtton KL. Neutrophil myeloperoxidase regulates T-cell-driven tissue inflammation in mice by inhibiting dendritic cell function. Blood (2013) 121:4195–204. 10.1182/blood-2012-09-456483 23509155

[17] de Kleijn S Langereis JD Leentjens J Kox M Netea MG Koenderman L. IFN-gamma-stimulated neutrophils suppress lymphocyte proliferation through expression of PD-L1. PLoS ONE (2013) 8:e72249. 10.1371/journal.pone.0072249 24015224 PMC3756078

[18] Bowers NL Helton ES Huijbregts RP Goepfert PA Heath SL Hel Z. Immune suppression by neutrophils in HIV-1 infection: role of PD-L1/PD-1 pathway. PLoS Pathogens (2014) 10:e1003993. 10.1371/journal.ppat.1003993 24626392 PMC3953441

[19] Langereis JD Pickkers P de Kleijn S Gerretsen J de Jonge MI Kox M. Spleen-derived IFN-gamma induces generation of PD-L1(+)-suppressive neutrophils during endotoxemia. J Leuko Biol. (2017) 102:1401–9. 10.1189/jlb.3A0217-051RR 28974543

[20] Hampton HR Chtanova T. The lymph node neutrophil. Sem Immunol. (2016) 28:129–36. 10.1016/j.smim.2016.03.008 27025975

[21] Maletto BA Ropolo AS Alignani DO Liscovsky MV Ranocchia RP Moron VG. Presence of neutrophil-bearing antigen in lymphoid organs of immune mice. Blood (2006) 108:3094–102. 10.1182/blood-2006-04-016659 16835380

[22] Gorlino CV Ranocchia RP Harman MF Garcia IA Crespo MI Moron G. Neutrophils exhibit differential requirements for homing molecules in their lymphatic and blood trafficking into draining lymph nodes. J Immunol. (2014) 193:1966–74. 10.4049/jimmunol.1301791 25015824

[23] Barnden MJ Allison J Heath WR Carbone FR. Defective TCR expression in transgenic mice constructed using cDNA-based alpha- and beta-chain genes under the control of heterologous regulatory elements. Immunol Cell Biol. (1998) 76:34–40. 10.1046/j.1440-1711.1998.00709.x 9553774

[24] Holmes K Lantz LM Fowlkes BJ Schmid I Giorgi JV. Preparation of cells and reagents for flow cytometry. Curr Prot Immunol. (2001) 5.3. 10.1002/0471142735.im0503s44 18432799

[25] Sanchez Vallecillo MF Ullio Gamboa GV Palma SD Harman MF Chiodetti AL Moron G. Adjuvant activity of CpG-ODN formulated as a liquid crystal. Biomaterials (2014) 35:2529–42. 10.1016/j.biomaterials.2013.12.002 24382332

[26] Hampton HR Bailey J Tomura M Brink R Chtanova T. Microbe-dependent lymphatic migration of neutrophils modulates lymphocyte proliferation in lymph nodes. Nat Commun. (2015) 6:7139. 10.1038/ncomms8139 25972253 PMC4479041

[27] Bardhan K Anagnostou T Boussiotis VA. The PD1:PD-L1/2 pathway from discovery to clinical implementation. Front Immunol. (2016) 7:550. 10.3389/fimmu.2016.00550 28018338 PMC5149523

[28] Jaillon S Galdiero MR Del Prete D Cassatella MA Garlanda C Mantovani A. Neutrophils in innate and adaptive immunity. Sem Immunopathol. (2013) 35:377–94. 10.1007/s00281-013-0374-8 23553214

[29] Abadie V Badell E Douillard P Ensergueix D Leenen PJ Tanguy M. Neutrophils rapidly migrate via lymphatics after Mycobacterium bovis BCG intradermal vaccination and shuttle live bacilli to the draining lymph nodes. Blood (2005) 106:1843–50. 10.1182/blood-2005-03-1281 15886329

[30] Chtanova T Schaeffer M Han SJ van Dooren GG Nollmann M Herzmark P. Dynamics of neutrophil migration in lymph nodes during infection. Immunity (2008) 29:487–96. 10.1016/j.immuni.2008.07.012 18718768 PMC2569002

[31] Tacchini-Cottier F Zweifel C Belkaid Y Mukankundiye C Vasei M Launois P. An immunomodulatory function for neutrophils during the induction of a CD4+ Th2 response in BALB/c mice infected with Leishmania major. J Immunol. (2000) 165:2628–36. 10.4049/jimmunol.165.5.2628 10946291

[32] Sagoo P Garcia Z Breart B Lemaitre F Michonneau D Albert ML. *In vivo* imaging of inflammasome activation reveals a subcapsular macrophage burst response that mobilizes innate and adaptive immunity. Nat Med. (2016) 22:64–71. 10.1038/nm.4016 26692332

[33] Hor JL Heath WR Mueller SN. Neutrophils are dispensable in the modulation of T cell immunity against cutaneous HSV-1 infection. Sci Rep. (2017) 7:41091. 10.1038/srep41091 28112242 PMC5253768

[34] Brackett CM Muhitch JB Evans SS Gollnick SO. IL-17 promotes neutrophil entry into tumor-draining lymph nodes following induction of sterile inflammation. J Immunol. (2013) 191:4348–57. 10.4049/jimmunol.1103621 24026079 PMC3795982

[35] Jakus Z Nemeth T Verbeek JS Mocsai A. Critical but overlapping role of FcgammaRIII and FcgammaRIV in activation of murine neutrophils by immobilized immune complexes. J Immunol. (2008) 180:618–29. 10.4049/jimmunol.180.1.618 18097064 PMC2647079

[36] Fossati G Bucknall RC Edwards SW. Insoluble and soluble immune complexes activate neutrophils by distinct activation mechanisms: changes in functional responses induced by priming with cytokines. Ann Rheum Dis. (2002) 61:13–9. 10.1136/ard.61.1.13 11779751 PMC1753889

[37] Aguilar JC Rodriguez EG. Vaccine adjuvants revisited. Vaccine (2007) 25:3752–62. 10.1016/j.vaccine.2007.01.111 17336431

[38] Billiau A Matthys P. Modes of action of Freund's adjuvants in experimental models of autoimmune diseases. J Leuko Biol. (2001) 70:849–60. 10.1189/jlb.70.6.849 11739546

[39] Teng TS Ji AL Ji XY Li YZ. Neutrophils and immunity: from bactericidal action to being conquered. J Immunol Res. (2017) 2017:9671604. 10.1155/2017/9671604 28299345 PMC5337389

[40] Kim MH Granick JL Kwok C Walker NJ Borjesson DL Curry FR. Neutrophil survival and c-kit(+)-progenitor proliferation in Staphylococcus aureus-infected skin wounds promote resolution. Blood (2011) 117:3343–52. 10.1182/blood-2010-07-296970 21278352 PMC3069674

[41] Pesce JT Liu Z Hamed H Alem F Whitmire J Lin H. Neutrophils clear bacteria associated with parasitic nematodes augmenting the development of an effective Th2-type response. J Immunol. (2008) 180:464–74. 10.4049/jimmunol.180.1.464 18097048 PMC2288648

[42] Culshaw S Millington OR Brewer JM McInnes IB. Murine neutrophils present Class II restricted antigen. Immunol Lett. (2008) 118:49–54. 10.1016/j.imlet.2008.02.008 18400308 PMC2430030

[43] Matsushima H Geng S Lu R Okamoto T Yao Y Mayuzumi N. Neutrophil differentiation into a unique hybrid population exhibiting dual phenotype and functionality of neutrophils and dendritic cells. Blood (2013) 121:1677–89. 10.1182/blood-2012-07-445189 23305731 PMC3591793

[44] Hulsdunker J Ottmuller KJ Neeff HP Koyama M Gao Z Thomas OS. Neutrophils provide cellular communication between ileum and mesenteric lymph nodes at graft-versus-host disease onset. Blood (2018) 131:1858–69. 10.1182/blood-2017-10-812891 29463561 PMC5909763

[45] Blomgran R Ernst JD. Lung neutrophils facilitate activation of naive antigen-specific CD4+ T cells during *Mycobacterium tuberculosis* infection. J Immunol. (2011) 186:7110–9. 10.4049/jimmunol.1100001 21555529 PMC3376160

[46] Chen X Oppenheim JJ. Contrasting effects of TNF and anti-TNF on the activation of effector T cells and regulatory T cells in autoimmunity. FEBS Lett. (2011) 585:3611–8. 10.1016/j.febslet.2011.04.025 21513711 PMC3164898

[47] Pillay J Kamp VM van Hoffen E Visser T Tak T Lammers JW. A subset of neutrophils in human systemic inflammation inhibits T cell responses through Mac-1. J Clin Invest. (2012) 122:327–36. 10.1172/JCI57990 22156198 PMC3248287

[48] Wang TT Zhao YL Peng LS Chen N Chen W Lv YP. Tumour-activated neutrophils in gastric cancer foster immune suppression and disease progression through GM-CSF-PD-L1 pathway. Gut (2017) 66:1900–11. 10.1136/gutjnl-2016-313075 28274999 PMC5739867

[49] Luo Q Huang Z Ye J Deng Y Fang L Li X. PD-L1-expressing neutrophils as a novel indicator to assess disease activity and severity of systemic lupus erythematosus. Arthr Res Ther. (2016) 18:47. 10.1186/s13075-016-0942-0 26867643 PMC4751645

[50] Luo Q Zeng LL Mei HY Huang ZK Luo ZQ Ye JQ PD-L1-expressing neutrophils as a novel indicator to assess disease activity of rheumatoid arthritis. Int J Clin Exp Med. (2017) 10:7716–24.

[51] Wang JF Li JB Zhao YJ Yi WJ Bian JJ Wan XJ. Up-regulation of programmed cell death 1 ligand 1 on neutrophils may be involved in sepsis-induced immunosuppression: an animal study and a prospective case-control study. Anesthesiology (2015) 122:852–63. 10.1097/ALN.0000000000000525 25437496

