# Supplementary material for: Neutrophils Which Migrate to Lymph Nodes Modulate CD4+ T Cell Response by a PD-L1 Dependent Mechanism
Source: Front Immunol. 2019 Jan 29;10:105. doi: 10.3389/fimmu.2019.00105 (PMC6362305; doi:10.3389/fimmu.2019.00105)
Supplement: Supplementary file 1 [file Data_Sheet_1.PDF]

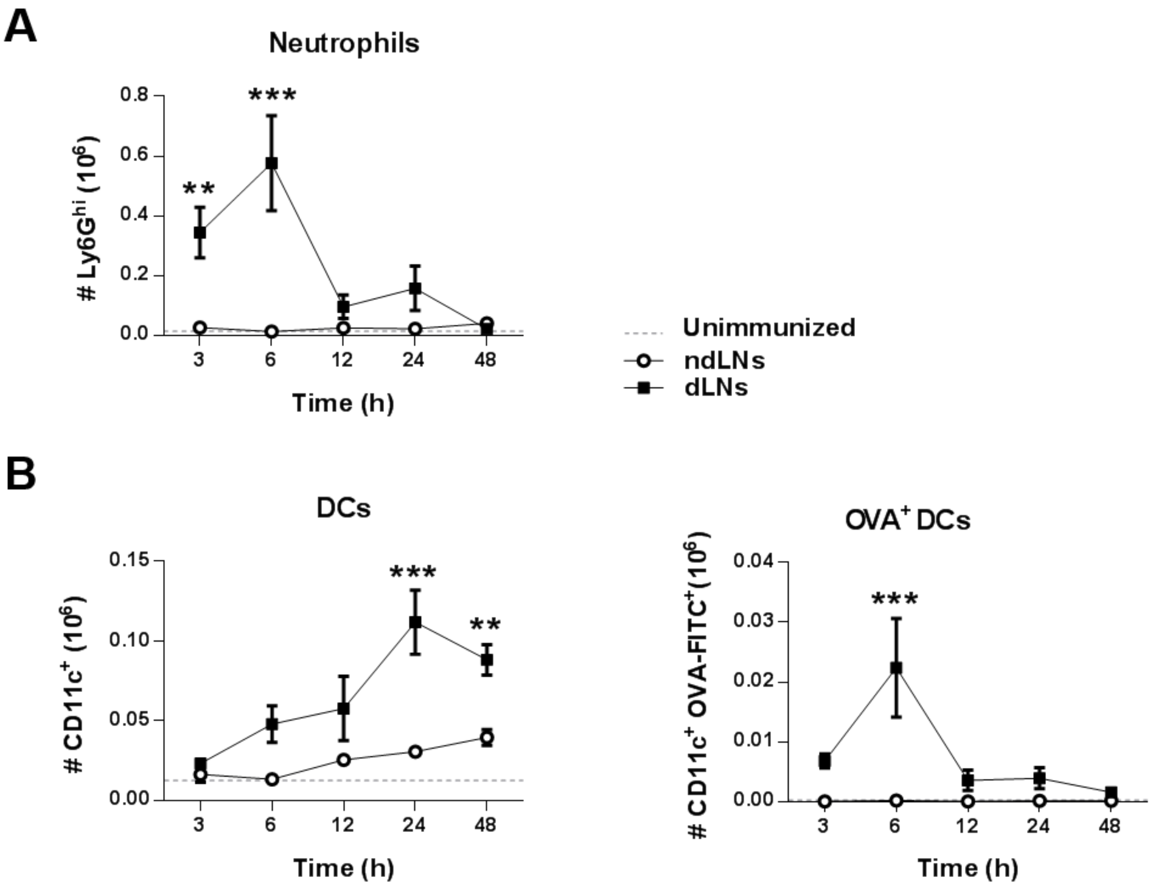

Supplementary Figure 1. Flow cytometry analysis of cell populations in dLNs and ndLNs obtained from immunized mice at different time points after footpad injection of OVA-FITC or SS, respectively. In the dotted line, normal values of LNs from unimmunized mice are shown as reference. Absolute number of **(A)** Ly6G^{hi} neutrophils and **(B)** CD11c^{+} DCs and OVA-FITC^{+} CD11c^{+} DCs. Results are representative of three independent experiments and are expressed as mean ± SEM (n= 4/group); **p<0.001, ***p< 0.001.

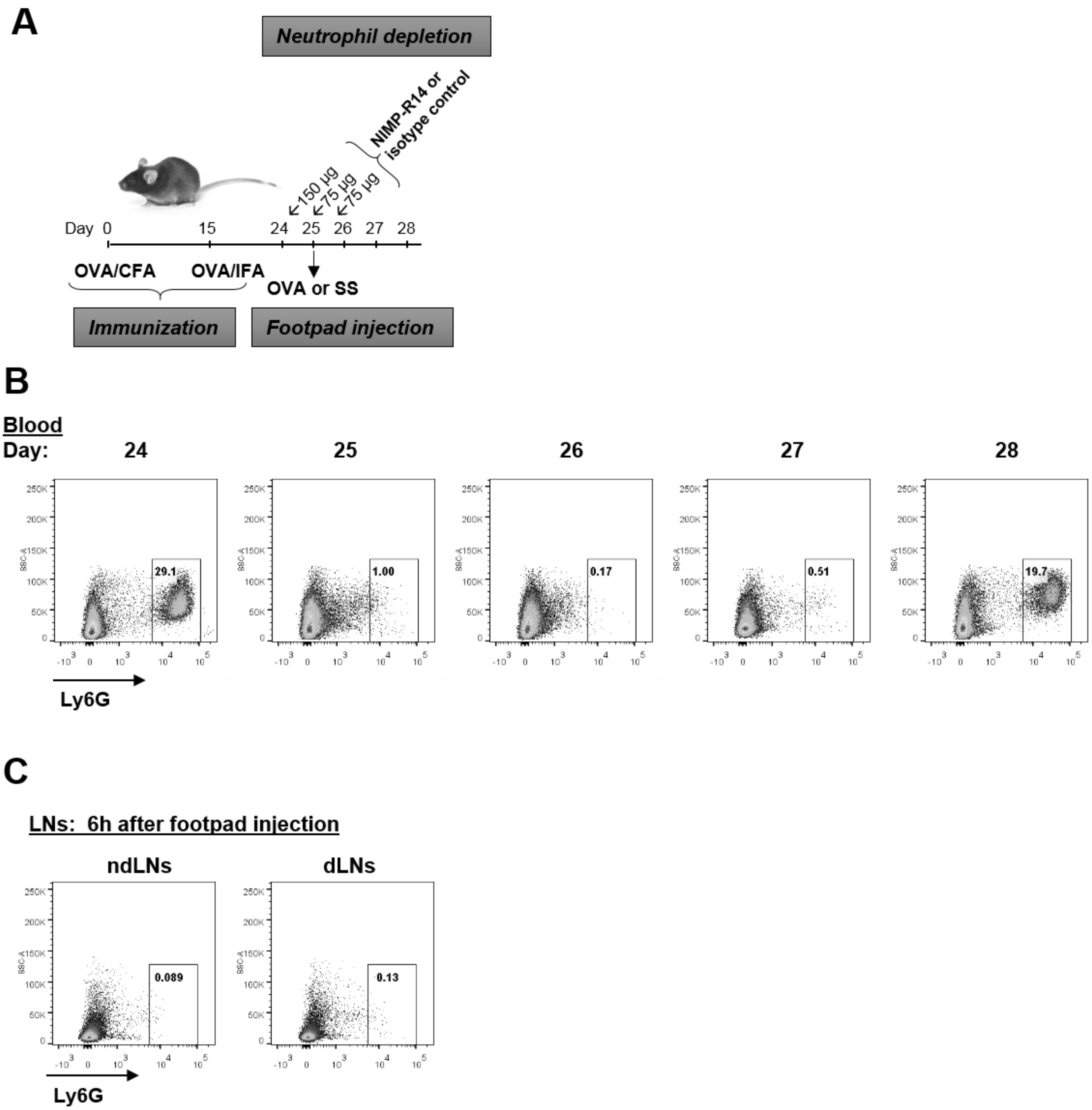

Supplementary Figure 2. (A) Schematic diagram of immunized mice treated with NIMP-R14 antibody to deplete neutrophils or isotype control. Flow cytometry analysis was performed in immunized mice treated with NIMP-R14 to control neutrophil depletion: neutrophils Ly6G^{hi} in **(B)** blood and **(C)** dLNs and ndLNs obtained at the indicated time. Results are representative of two independent experiments and are expressed as representative dot plots with numbers indicating percentage of gated cells.

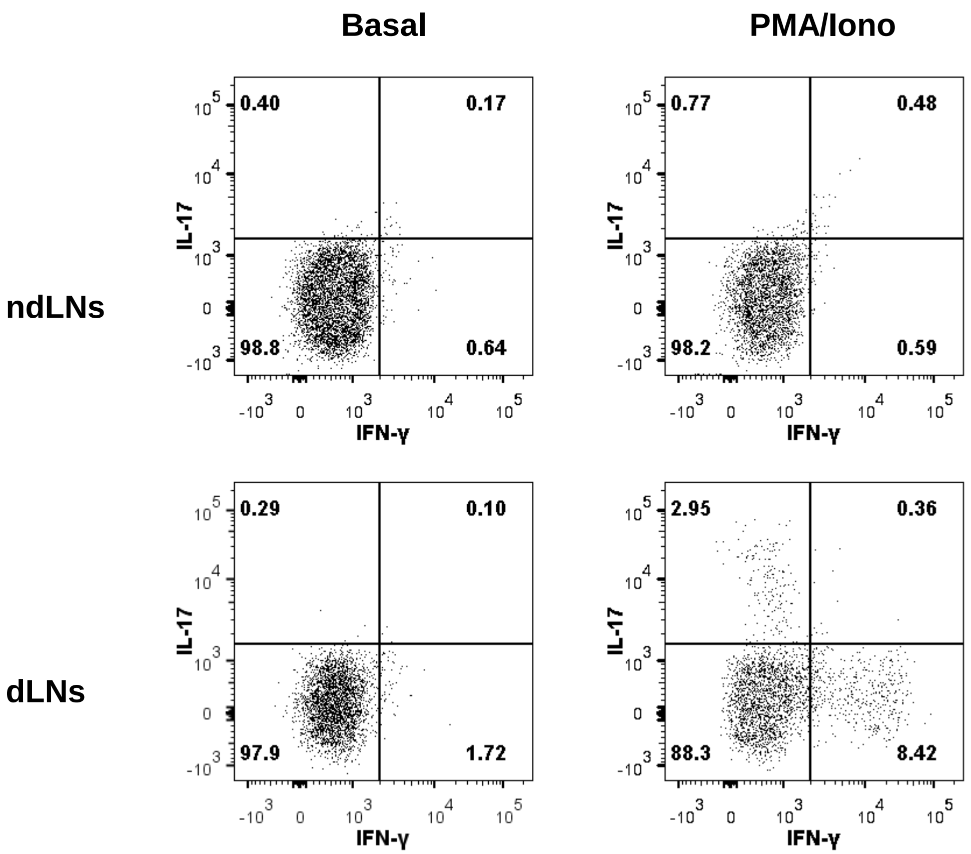

Supplementary Figure 3. dLNs and ndLNs cells were obtained from immunized mice 24 h after OVA or SS footpad injection. Cells were cultured for 72 h with PMA and Ionomycin or medium alone (basal) and later the percentage of IL-17$^+$ and IFN-γ^+ on CD4$^+$ T cells was determined by flow cytometry analysis. Representative dot plot with number indicating percentage of cells. Results are representative of three independent experiments and are expressed as mean ± SEM (n= 4-5/group).

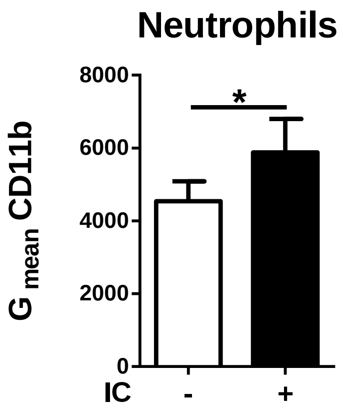

Supplementary Figure 4. Geometric mean (Gmean) of CD11b levels in neutrophils purified from BM as positive control of IC-stimulation of neutrophils. Neutrophils were stimulated for 1 h with previously formed OVA/anti-OVA IC. Results are representative of three independent experiments and are expressed as mean ± SEM (n= 4-5/group); *p<0.05.
